# Transactions of the Buffalo Obstetrical Society

**Published:** 1885-05

**Authors:** 


### Transactions of the Buffalo Obstetrical Society.

*Stated Meeting, March 24, 1885.*

The President, Dr. W. W. Potter, in the Chair; Dr. William H. Thornton, Secretary.

Dr. J. W. Keene read a paper on "The Use and Abuse of the Tampon in Abortion."

He took the ground that the tampon was often injudiciously employed in threatened abortions, and thus frequently precipitated the catastrophe it was intended to avert.

Various authors were quoted, all agreeing that the tampon was not only capable of exciting uterine contractions, but that it invariably did so, and was, moreover, employed by abortionists for the purpose of inducing miscarriage.

The indications for the tampon unanimously agreed upon by many of the authors cited were two: First, when the abortion was inevitable; and second, when the hemorrhage imperiled the woman's life or endangered her health.

We should not, he said, be too ready to regard the abortion as inevitable; although many practitioners, and some of the authors, appear to consider every abortion that threatens as inevitable. Playfair states that abortion is inevitable, when the os is found beginning to open so that the finger can pass through it and touch the ovum, "especially if pains also exist." Other authorities were cited far more cautious in pronouncing an abortion inevitable.

The essayist then cited some of the instances recorded, where, in cases apparently past all hope, the abortion had not occurred and the woman had gone to term. Hence he thought that all doubtful cases should be treated as hopeful rather than as inevitable. If the fœtus was known to be dead its speedy expulsion was, of course, to be desired; and the tampon was a safe and ready means with which to excite the necessary uterine action. But the diagnosis of death, prior to the fifth month, could be made with certainty in but few, very few, cases. The actual expulsion of the fœtus was about the only positive proof of its death, and, so long as its death was problematical, we should proceed as though it were known to be living. The reader thought Schroeder's view the safest one, viz.: "The hope of preventing the abortion should not be abandoned unless a part of the ovum has already passed out of the external os."

The second indication for the application of the tampon, viz., hemorrhage compromising the safety of the woman, the essayist thought, had quite as wide a range of latitude in its interpretation as the first. A hemorrhage which one physician would deem alarming, might be, in the opinion of another, a matter of small moment. It was here that the judgment of the physician must be his guide. Experience, idiosyncrasy, and the personal equation inevitably affect his judgment. The authorities differ, as was shown by citations from several works on obstetrics. Schroeder holds that bleeding may go on in these cases "to the highest degree of anæmia, syncope, and pulselessness, but is very seldom followed by death," nor is the woman's health permanently impaired thereby. Others would plug for a very slight hemorrhage. The proper course was, no doubt, the middle one between these extremes.

The reader deprecated the routine practice of plugging, merely as a preliminary measure, in threatened abortion with comparatively insignificent bleeding, and concluded his paper by saying: "Let it be laid down as an axiom, that the tampon is legitimately employed only when, for good and sufficient reasons, it is necessary to terminate gestation."

*Discussion*—Dr. R. L. Banta said that he had not observed such great danger of producing abortion with the tampon as the author of the paper would have us believe. He was formerly in the habit of using the tampon whenever he found a woman losing a thimbleful of blood, but of late he had not as great fear of this sort of hemorrhage. In one of his recent cases he found the woman flowing profusely, and presumed the abortion inevitable. After watching the case awhile he went away for an hour, and upon his return found the flow steadily going on. He then gave a full dose of morphine and went home. The next morning the hemorrhage had ceased, and he now thought gestation would continue to term.

Dr. C. C. Frederick, in reviewing the various methods of tamponing the vagina for hemorrhage, advocated the employment of the colpeurynter of Braun as an efficient, as well as an easy and cleanly way of performing this operation. One of these rubber bags could easily be introduced into the vagina and inflated so as to completely dam up the flow; it could also be readily withdrawn, with little disturbance to the patient, when necessary to make an examination. He much preferred this method to any other, since he had become familiar with it, as neater, prompter, and more convenient.

Dr. Charles G. Stockton had been rather frequently called upon to consider whether or not he should use a tampon. The question of using the tampon arises at other times than that mentioned in the paper; for instance, when there is, after the escape of the fœtus, retention of the secundines. Many practitioners, he observed, were accustomed to use the tampon then, and he thought the dangers which were supposed to follow the retention of the secundines involved questions worthy of discussion in this connection. He was surprised the other day when a young physician said to him that he never removed the secundines, unless late in pregnancy; that they always came away spontaneously, and that his patients never had septicæmia or other untoward conditions, mentioning something like one hundred or more cases that he had attended within a comparatively short time. This seemed surprising to him, and he would like to ask the members what their habit was in regard to the removal of the secundines in abortion cases.

Dr. M. Hartwig thought that if the tampon were used at all the indications for its employment were correctly given in the paper. He was of the opinion that tampons should be given up almost entirely. Although he used them in some cases he fully believed, as Schroeder states, that a woman will not bleed to death from abortion before the end of the fourth month. It was important to distinguish between the use of the tampon during pregnancy or after confinement, and in cases which have nothing to do with pregnancy—uterine bleedings from other causes. He should say that the latter cases were the only ones where he might use the tampon. The old-fashioned kite-tail tampon would certainly cause contraction of the womb. So, too, would the colpeurynter, if it was blown up sufficiently to check the bleeding. There were other ways of tamponing, as with compressed sponge; another method was to tampon the mouth of the womb through the speculum. This was the method he preferred if he used any. But, all told, in cases of bleeding during pregnancy or after the expulsion of the fœtus, he had decidedly come to the conviction that the tampon was unneccessary; because, if the hemorrhage was insignificant the woman could stand it; small doses of ergot, or even large doses—which never produce abortion of themselves—would usually check the flow. As a scientific ruling, he would put forth the idea that the tampon should not be used at all in these cases. The question of the viability of the fœtus ought to be decided with a good deal of probability. If the woman had bled considerably, and the os was patulous, it was safe to conclude that the fœtus was dead.

Dr. W. S. Tremaine was very glad to hear the foregoing views expressed. Of late he had given up the practice of obstetrics except in consultation, though some years ago he did a good deal in this way. He never used a tampon but once, and never would again if he could avoid it. He deemed it one of the most barbarious, unsurgical, and nonsensical things that was ever invented. Dr. Hartwig had expressed about his ideas in regard to it. He used to see a good deal of hemorrhage, and he generally succeeded in controlling it with alum-water, which was thrown up into the uterus. He had never seen a death from post partum hemorrhage. When the bleeding was at all copious, abortion was inevitable; but about the surest way to bring it on was to give opium.

Dr. Thomas Lothrop could not understand how gentlemen who practice obstetrics, and have aborting women to care for, could get on well in their management of these cases, especially in the second and third months of pregnancy, without the employment of the tampon. He looked upon its use as an absolute necessity in many instances, and he criticised any method of practice which permitted women to lose large quantities of blood, when it could be controlled with a simple plug. It was seldom necessary to use the tampon until after embryonic life was either extinct, or certain to become so, whatever was done. When he had felt compelled to use the tampon, it had almost always been in retention of the secundines after expulsion of the fœtus. His method of using it was to place the patient in the Sims' position, and, with the aid of the Sims' speculum, to pack the vagina tightly with cotton pledgets. As for septic infection, he had never had any such trouble following the tampon in his practice. There are two symptoms in abortion which he considered important to combat, viz., pain, and hemorrhage, and the two agents which he recommended for this purpose, above and beyond all others, were opium, and the tampon.

Dr. Alfred C. Girard (present by invitation) said that he was willing to give his opinion, since he had been so kindly asked to join in the discussion, for simply what it was worth when thrown into the scale. He had seen and treated a good many cases of hemorrhage of the character in question, and had tried many means to check it. When called to such a case, if he found the os partially dilated and the cervical portion softened, he was satisfied that the fœtus was dead, and he felt, under such circumstances, that the best mode of procedure was to bring on rapid uterine contraction, expecting thereby to prevent further hemorrhage. In following this rule he always felt that he had done the best he could for the patient. He believed that the tampon would, in nearly every case, bring on an abortion, so he would not advise its use when there was any reasonable hope of conducting the case to term. When, however, the physician was satisfied that the fœtus was dead, he conld not see how he could do better than to employ the tampon, if the hemorrhage was persistent.

The President inferred that the general import of the paper was against the use of the tampon. He wished to take exception to that teaching. He had, during twenty-six years of practice, used the tampon constantly when needed, and had never yet had occasion to regret it. He employed the tampon for two purposes: First, in extreme cases, to save life when hemorrhage was alarming; second, to avoid the loss of blood in a class of cases where the flow, though not copious, could not be well borne for constitutional reasons. In short, he considered it the physician's duty to economize the loss of blood—to reduce it to the very minimum—in these days of anæmia, neurasthenia, and mal-nutrition. He was sorry to hear gentlemen speak so slightingly of what they were pleased to term the "loss of a little blood." They asserted, with a grandiose air, that they were not afraid to see their patients bleed, that women could stand the loss of great quantities of blood, and yet recover. Did they recover? Was it not true that many such cases make sad work in getting up? Between anæmia, neurasthenia, and ill-nourished tissues on the one hand, and subinvolution, cellulitis, and displacements on the other, they presented a pitiable picture of invalidism, which a little more care and thoroughness in early management might sometimes have averted.

He thought that failures in the employment of the tampon were due to its imperfect adjustment. In case of violent hemorrhage it could certainly be controlled by placing the woman in the Sims' position, and packing the vagina tightly, so tight, indeed, that there could be no bleeding. In case of hemorrhage, after the escape of the fœtus, the secundines, or a portion thereof, being undelivered, he would make the attempt to remove them if the bleeding was not violent. If, however, it was copious, he would tampon in the manner just mentioned. He would not hazard the further loss of blood, when he had such an efficient agent at hand to control it. He believed in the curette, and would employ it to remove any tags or shreds of the placenta yet hanging to the uterine walls, after which it would be good practice to brush over the cavity with Churchill's tincture of iodine.

